# Social inclusion increases with time for zero-tillage wheat in the Eastern Indo-Gangetic Plains

**DOI:** 10.1016/j.worlddev.2019.06.006

**Published:** 2019-11

**Authors:** Alwin Keil, Archisman Mitra, Amit K. Srivastava, Andrew McDonald

**Affiliations:** a CIMMYT-India, CG Block, National Agricultural Science Centre (NASC) Complex, DPS Marg, New Delhi 110012, India; b CIMMYT-Nepal, Nepal Agricultural Research Council (NARC) Agricultural Botany Division, Khumaltar, Kathmandu, Nepal

**Keywords:** Zero-tillage adoption, Service economy, Social inclusiveness, Social network effects, South Asia, Bihar

## Abstract

Sustainable intensification (SI) approaches to agricultural development are urgently needed to meet the growing demand for crop staples while protecting ecosystem services and environmental quality. However, SI initiatives have been criticized for neglecting social welfare outcomes. A recent review found that better-off farmers benefitted disproportionately from SI and highlighted the dearth of studies assessing the equity of outcomes. In this study, we explore the social inclusiveness of zero-tillage (ZT) wheat adoption in Bihar, India. ZT is a proven SI technology for enhancing wheat productivity while boosting profitability and reducing greenhouse gas emissions from agricultural machinery in the densely populated Indo-Gangetic Plains. With an average landholding size of 0.39 ha, most farmers in Bihar depend on custom-hiring services to access the technology. While service provision models should foster inclusive growth by reducing financial barriers to technology adoption, early evidence suggested that smallholders remained at a disadvantage. Building on this previous research, we use a panel dataset from 961 wheat-growing households that spans a six-year period to analyze ZT adoption dynamics over time while accounting for the role of social networks and access to service provision. Using a heckprobit approach to correct for non-exposure bias, we compare determinants of ZT awareness and use in 2012 and 2015. We apply a multinomial logit model to identify determinants of early adoption, recent adoption, non-adoption, and dis-adoption. Furthermore, we explore the quality of ZT services as an additional dimension of socially-inclusive technology access. We find that the strong initial scale bias in ZT use declined substantially as awareness of the technology increased and the service economy expanded. Land fragmentation replaced total landholding size as a significant adoption determinant, which also affected the quality of ZT services received. Hence, farmers with small but contiguous landholdings appear to have gained a significant degree of access over time. We conclude that early-stage assessments of SI may be misleading, and that private sector-based service provision can contribute to socially inclusive development outcomes as markets mature.

## Highlights

• Farmers in Bihar access zero-tillage (ZT) technology mostly via custom-hiring services.
• Scale bias in ZT use declined as farmer awareness increased and ZT services expanded.
• Land fragmentation replaced landholding size as a significant adoption determinant.
• Farmers with small but contiguous landholdings have gained access to quality ZT services.
• Private sector-based mechanization service provision can lead to socially inclusive outcomes.

## 1 Introduction

Global food demand is growing rapidly due to population growth and increased consumption of resource-intensive meat and dairy products resulting from rising per-capita incomes (Garnett et al., 2013, Godfray et al., 2010). Refining earlier estimates by Tilman et al., 2011, Alexandratos and Bruinsma, 2012, Hunter et al., 2017 project a 26–68% increase in current cereal production that will be required to meet global demand in 2050. This increase needs to be achieved in the context of growing competition for land, water and energy resources, with climate change posing additional challenges (Garnett et al., 2013, Godfray et al., 2010). At the same time, the environmental footprint of agriculture, such as nutrient losses and greenhouse gas (GHG) emissions, need to be reduced dramatically in order to protect critical ecosystem functions (Hunter et al., 2017, Tilman et al., 2011). Land clearing to increase agricultural production through area expansion results in far greater GHG emissions than the intensification of production on existing croplands (Tilman et al., 2011). Hence, closing yield gaps on existing cropland is essential if the growing food demand is to be met with minimal environmental consequences, necessitating ‘sustainable intensification’ (SI) approaches to crop management (Garnett et al., 2013). A practice is defined to be ‘sustainable’ if it is economically viable, environmentally sound, and socially inclusive (UN General Assembly, 2005). Contemporary SI initiatives have been criticized for being too narrowly focused on production while neglecting environmental (Hunter et al., 2017) and social welfare outcomes (Garnett et al., 2013, Loos et al., 2014, Rasmussen et al., 2018). Improving the latter is contingent not only on demonstrating measurable gains at the farm household level on the basis of pilots and proofs of concept, but also on accelerating socially inclusive adoption at scale.

The Indo-Gangetic Plains (IGP) are home to >20% of the global population, and sustainably enhancing the productivity of the prevailing rice-wheat cropping systems is vital for ensuring future food security in South Asia (Chauhan, Mahajan, Sardana, Timsina, & Jat, 2012). The potential to increase yields is particularly large in the Eastern IGP, such as the state of Bihar. On the one hand, Bihar has the lowest wheat yields in the IGP, averaging 2.17 MT ha^−1^ over the period 2010/11–2015/16, a mere 46% of the 4.70 MT ha^−1^ achieved in the Northwestern state of Punjab (MoA, 2017). Furthermore, at 1106 persons km^−2^, Bihar is extremely densely populated (total population 104 million), resulting in average landholding sizes of 0.39 ha, whereas farmers in Punjab have on average 3.77 ha available for cropping (all-India average 1.15 ha; MoA, 2017, based on Agricultural Census 2010–11); 91% of Bihari farmers have less than one hectare of arable land, thus falling into the ‘marginal’ category according to the Indian farm size classification (MoA, 2017). On the other hand, the Eastern IGP has a wealth of under-developed water resources (Aggarwal et al., 2004, DoA, 2008), whereas excessive irrigation has led to dramatic declines in groundwater tables in the Northwest (Humphreys et al., 2010, chap. 5). To meet both state and national-level cereal demand over the coming decades, technologies are urgently needed that sustainably enhance agricultural productivity in the Eastern IGP and are adoptable at scale by smallholders with landholding sizes well below one hectare.

The use of zero tillage (ZT) in wheat in combination with residue retention has demonstrated clear agronomic and economic benefits, while improving the environmental footprint of agriculture (Aryal et al., 2015, Chauhan et al., 2012, Erenstein and Laxmi, 2008, Gathala et al., 2013, Mehla et al., 2000). The prevailing ZT practice uses a zero-till drill attached to a relatively small four-wheel tractor^1^ to sow wheat directly into unplowed fields with a single pass (Erenstein & Laxmi, 2008). The typical ZT drill opens 6–13 narrow slits using inverted-T openers to place both seed and fertilizers at a depth of 7.5–10 cm (Mehla et al., 2000). In contrast, conventional-tillage in wheat typically involves multiple passes of the tractor to accomplish plowing, harrowing and planking before sowing (Erenstein & Laxmi, 2008).

Reviewing the impacts of ‘conservation tillage’, mostly ZT, on wheat productivity across South Asia, Krishna, Keil, Aravindakshan, and Meena (2017) found that 13 out of 25 published studies reported a significant yield gain, while none indicated a statistically significant yield loss. While most of the studies were conducted in the Northwestern IGP, based on a random sample of 1000 farm households Keil, D'souza, and McDonald (2015) found that in Bihar the use of ZT – with only partial residue retention – led to a yield increase of 498 kg ha^−1^ (19%) over conventional-tillage wheat. In addition, the practice reduced crop establishment costs by 46%. The yield gains and cost savings amounted to a combined average economic benefit of 110 US$ ha^−1^, equivalent to a 6% increase in annual household income. The ZT-induced yield gain estimated by Keil et al. (2015) was very similar to that reported from on-farm trials in Bihar (490 kg ha^−1^; Dhiman, Kumar, & Om, 2003). In addition to providing monetary gains to farmers, the use of ZT in wheat has been shown to enhance water use efficiency (Erenstein & Laxmi, 2008), improve soil physical properties (Singh, Phogat, Dahiya, & Batra, 2014), and reduce GHG emissions (Aryal et al., 2015).

Despite the proven benefits of the technology, the adoption of ZT wheat has remained slow, with the highest rate estimated at 25%^2^ in the Northwestern IGP, and the lowest, 2%, in the East (Singh, Erenstein, Saharawat, Chaudhary, & Jat, 2012). The observed regional differences in the uptake of ZT seemed to be closely associated with the time of introduction of the technology (ibid.). In the Eastern IGP in particular, the above-mentioned small landholding sizes complicate the uptake of the technology as tractor and ZT drill ownership is not a tenable goal for the large majority of farmers; hence, they rely on custom-hiring services to access the technology (Erenstein and Laxmi, 2008, Keil et al., 2016). Furthermore, compared with the Northwestern IGP, farmers’ awareness of the technology is still low (Keil et al., 2017, Singh et al., 2012).

Taking these factors into account, Keil et al. (2017) assessed determinants of ZT wheat adoption in Bihar using cross-sectional data collected in 2013 and found a distinct scale bias, with larger, better educated, and higher-caste farmers being more likely to know about and use the technology. This is in line with the critique of SI initiatives articulated by Rasmussen et al. (2018): in their recent review of literature evaluating 60 cases of agricultural intensification, the authors found that well-being impacts tended to be unevenly distributed, favoring better-off farmers. However, the authors emphasized that only few studies examined socially disaggregated outcomes, which they identified as an important research gap. For the case of ZT wheat in Bihar, Keil et al. (2017) hypothesized that the scale bias may (in part) be due to the business logic of custom-hiring services, where larger-scale farmers were more attractive customers for the relatively few ZT service providers (SPs) in the area. As is the case with most technology adoption studies, Keil et al. (2017) present a snapshot in time and acknowledge that the technology was in a nascent stage of diffusion. A potential weakness of this and other cross-sectional adoption studies is that technology users may erroneously be classified as ‘adopters’ although they may only be testing and possibly discontinuing the practice (Doss, 2006). Furthermore, later adopters may systematically differ from very early adopters both with respect to asset endowment and less observable characteristics, such as risk preferences and innovativeness (Rogers, 2003).

We contribute to filling the knowledge gap on the thus-far neglected social dimension of SI uptake and address the shortcomings of cross-sectional technology adoption studies with a unique panel dataset that allows us to examine adoption *dynamics* of ZT wheat in Bihar over time. With the overall goal of deriving lessons for SI initiatives in developing countries in general, the objectives of this paper are (1) to assess the development of the social inclusiveness of ZT awareness and use; (2) to examine how the determinants of ZT awareness and use have changed over time; and (3) to identify influencing factors of early adoption, recent adoption, non-adoption, and dis-adoption; (4) in addition, for a knowledge intensive technology which is accessed mostly via custom-hiring services, the *quality* of service received constitutes another crucial dimension of socially inclusive access. We therefore explore the quality of custom-hiring services for ZT and its influencing factors.

Salient features of this paper are as follows: (i) information on ZT use covering a six-year period allows us to classify farmers according to objective (3) above; (ii) geo-referenced census data of ZT SPs in the area allow us to explore the effect of physical proximity of service providers on farmers’ knowledge and use of the technology (over time); (iii) as ZT is a knowledge intensive technology and still relatively new to the research area, we use a two-stage estimation procedure that corrects for non-exposure bias; (iv) acknowledging the importance of access to information in the early stage of technology diffusion, we explore the role of farmers’ social networks in the diffusion process (over time), allowing for both endogenous and exogenous network effects; and (v), to the best of our knowledge, this is the first study to explore the quality of mechanization services and its determinants, which is a crucial dimension to consider in a case where a knowledge intensive technology is accessed mostly via custom-hiring services.

The remainder of the paper is organized as follows: Section 2 provides a brief description of the research area, the sampling approach, and the kind of data used for the analysis; Section 3 derives our econometric model estimation strategy and details the final model specifications; Section 4 presents and discusses the findings from our descriptive and econometric analyses, while Section 5 concludes and derives general lessons for SI related research and extension in developing countries.

## 2 Research area, sampling procedure, and data collection

**Fig. 1:**
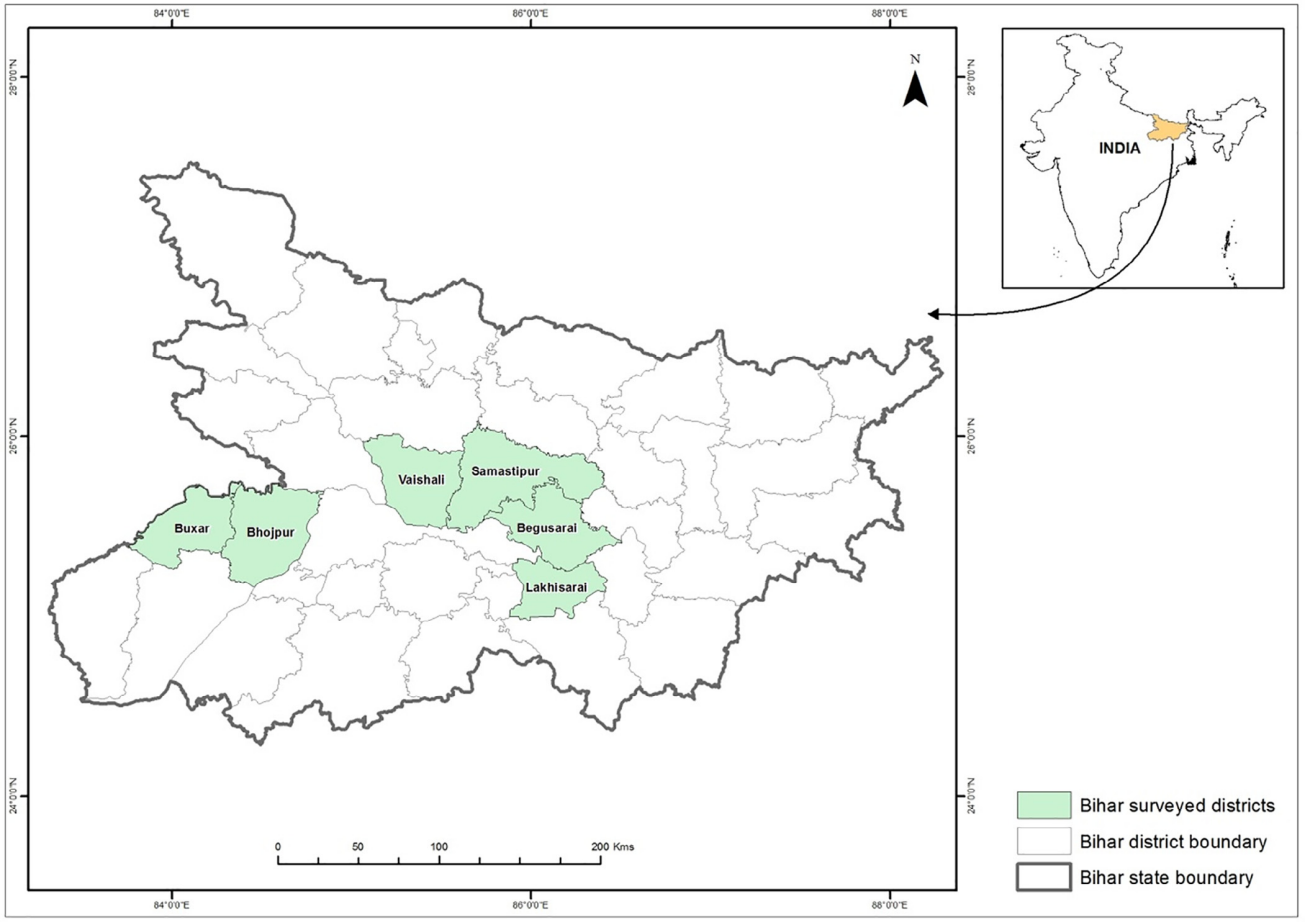
Map of the state of Bihar in northern India, highlighting the survey districts.

Agriculture is the main occupation in Bihar with almost 81% of its population engaged, whereas its contribution to the state’s domestic product is 42% (DoA, 2008). Paddy, wheat, pulses, maize, potato, sugarcane, oil seeds, tobacco and jute are the principal crops grown. ZT is almost exclusively used in wheat which is grown in the winter (*rabi*) season, extending from November to early April. Although Bihar is endowed with good soil, sufficient rainfall and abundant groundwater, its agricultural productivity is one of the lowest among Indian states (DoA, 2008). The research area is composed of six districts where the Cereal Systems Initiative for South Asia (CSISA; www.csisa.org) has focused research and out-scaling activities for sustainable intensification technologies since 2009 (Fig. 1). Using a cluster sampling approach, a first round of survey was conducted in 2013 among a random sample of 1000 wheat growing farm households in 40 villages. Owing to the nascent stage of ZT diffusion in the area, the village-level sampling frame was confined to 87 villages with at least 10 ZT users in the target districts, as documented by CSISA; furthermore, the sample was stratified by ZT adoption status to ensure an adequate size of the adopter sub-sample. The household-level sampling frame was compiled through a brief census survey within the 40 randomly selected villages, in which wheat growing farmers were identified and their ZT adoption status elicited. In 2016, a second round of survey was conducted among the same sample households. Thirty-nine households (3.9%) could not be re-interviewed due to prolonged absence or permanent migration, resulting in a sample of 961 households for which panel data are available. The primary focus of the data collected for our analysis were the growing seasons directly preceding the two rounds of survey, i.e. *rabi* seasons 2012/13 and 2015/16. However, recall data on ZT use in the two previous *rabi* seasons were also collected in each survey round, resulting in data covering the six-year period from 2010/11 through 2015/16^3^ [dataset] (Keil, Mitra, D'souza, & McDonald, 2018a).

Apart from eliciting information on asset endowment and farming practices, survey respondents were also asked to provide basic information on three farmers with whom they interacted most frequently about agricultural issues in order to be able to capture potential individual social network effects on ZT adoption. In addition to the household survey data, GPS positions of ZT service providers who were active in the area were available from CSISA. The geographic coordinates of service providers were integrated into our modeling approach as an additional potential determinant of ZT adoption.

Apart from the farm household survey, a census survey of 245 ZT service providers operating in the six target districts was conducted in 2013, and the same SPs were revisited in 2016 [dataset] (Keil, Mitra, D'souza, & McDonald, 2018b). For our analysis of quality determinants of mechanization services it was possible to match a subset of ZT users among the sample farm households (N = 48) with SPs in our dataset, allowing the inclusion of SP characteristics as regressors in the analysis. Hence, a salient feature of all regression models presented in this paper is the inclusion of regressors that are not based on the primary observation units (survey households), but on their social network members and ZT service providers. All data were collected from household heads by professional enumerators through structured interviews using CAPI software.^4^

## 3 Methodological approach

### 3.1 Model estimation strategy

#### 3.1.1 Accounting for non-exposure to the ZT technology

Following the approach of van de Ven and van Praag (1981) and building on Keil et al. (2017), we apply a two-stage estimation framework using a probit model with sample selection to account for the fact that farmers who do not know about ZT technology have no chance to adopt the practice. The first-stage probit model identifies determinants of ’knowledge exposure’ which implies that the farmer understands the attributes of the technology (Kabunga, Dubois, & Qaim, 2012); in the second stage, a probit model identifies determinants of technology adoption among the aware sub-sample. Since the selection process is non-random, non-exposure bias needs to be controlled for (Diagne & Demont, 2007). This is achieved by including the Inverse Mills Ratio (IMR) in the second-stage probit model, analogous to the method proposed by Heckman (1979) for the case of a second-stage regression with a continuous dependent variable (‘heckprobit’ model).

#### 3.1.2 Accounting for social network effects in the adoption process

Since the seminal paper by Feder, Just, and Silberman (1985) on the adoption of agricultural innovations, which considered individual-specific farm and farmer characteristics as potential adoption determinants, micro-level adoption studies have been extended to include more dynamic elements related to social learning (Feder and Savastano, 2006, Foster and Rosenzweig, 1995, Granovetter, 2005). Hereby, farmers may not only be influenced by the adoption behavior of their individual social networks (endogenous network effect), but also by their network members’ characteristics, such as age, education, and caste (exogenous network effect), as differentiated by Manski (2000). Drawing on the approach of Matuschke and Qaim (2009), we account for endogenous and exogenous individual network effects as in the following equation:

(1) $$y_{\mathrm{it}}={\beta X}_{\mathrm{it}}+{\delta y}_{n\left(it\right)}+{\varepsilon X}_{n\left(it\right)}{+\theta {\mathrm{SP}}_{\mathrm{it}}+u}_{\mathrm{it}}$$

where $y_{\mathrm{it}}=1$ if the household uses ZT in time period *t*, and $y_{\mathrm{it}}=0$ otherwise. As pointed out by Feder et al. (1985), a binary yes/no specification of the outcome variable has severe shortcomings in instances where there is great variation in the adoption *intensity*; i.e., a binary variable would not differentiate between farmers who used a new technology on their entire cultivated area and those who tested it on a small portion of their land. However, in the case of ZT wheat we find that, once the decision is made to use ZT, the practice is used on the entire wheat area by >82% of adopters, justifying the use of a binary dependent variable. Further, $X_{\mathrm{it}}$ is a vector of exogenous regressors, $y_{n\left(it\right)}$ denotes the adoption behavior of household *i*’s individual social network, $X_{n\left(it\right)}$ is a vector of exogenous network member characteristics, and ${\mathrm{SP}}_{\mathrm{it}}$ is a measure of the density of ZT service providers around household *i*; β,δ,ε and θ are (vectors of) parameters to be estimated, and $u_{\mathrm{it}}$ is a random error term. However, adopting the approach of Keil et al. (2017), we extend the methodology of Matuschke and Qaim (2009) by accounting for potential non-exposure bias as elaborated above. Hence, we estimate the model:

(2) $$y_{\mathrm{it}}=\dot{\beta }{\dot{X}}_{\mathrm{it}}+\beta_{\lambda }{\dot{\lambda }}_{\mathrm{it}}+u_{1it}$$

where ${\dot{X}}_{\mathrm{it}}$ encompasses all regressors included in Eq. (1) and ${\dot{\lambda }}_{\mathrm{it}}$ is the IMR derived from an exposure equation of the form

(3) $$a_{\mathrm{it}}=\dot{\gamma }{\dot{Z}}_{\mathrm{it}}+u_{2it}$$

where ${\dot{Z}}_{\mathrm{it}}$, in addition to other regressors, contains endogenous and exogenous individual social network characteristics as specified in Eq. (1). Extending the study by Keil et al. (2017), we estimate two separate models for the wheat growing seasons 2012 and 2015 to explore how the factors influencing awareness and use of ZT changed over time within this early stage of ZT diffusion in the research area^5^. We use the Stata 13 software package (www.stata.com) to estimate the ‘heckprobit’ model, specifying heteroskedasticity-consistent standard errors that account for clustering of the sample at the village level. The model produces a Wald test on the null hypothesis that the correlation between error terms $u_{1it}$ and $u_{2it}$ *ρ* = 0,^6^ in which case non-exposure bias would not exist, and Eq. (2) would simplify to Eq. (1).

#### 3.1.3 Accounting for timing and continuity of ZT adoption

The merit of the heckprobit models outlined above is the differentiation of factors affecting awareness and use of ZT, correcting for non-exposure bias. However, these models do not clearly disentangle factors affecting ZT use among early adopters (those who used the technology in 2012 or earlier) and those that influenced its use in more recent times, as the 2015 model treats all ZT users the same, no matter when they started using the technology. Nor does the model consider farmers who used ZT at an earlier stage but discontinued the practice before 2015 any different than farmers who never used the technology. Because of these shortcomings we estimate a multinomial logit model (cf. Wooldridge, 2006) that takes the timing and continuity of ZT use into account and estimates the probabilities of four outcomes, namely ‘early adoption’ (continuous use of ZT since 2012 or earlier), ‘recent adoption’ (continuous use since 2013 or later), ‘non-adoption’ (no use of ZT over the period 2010–2015), and ‘dis-adoption’ (at least three consecutive years of non-use after initial use of ZT or explicit statement of having discontinued the practice).

#### 3.1.4 Accounting for the quality of ZT services received

For a knowledge intensive technology that is accessed mostly via custom-hiring services, the *quality* of the service received is a crucial dimension of social inclusiveness of technology access. For instance, *timely* wheat establishment is of particular importance in the Eastern IGP to avoid yield depression due to terminal heat stress (Chauhan et al., 2012, Erenstein and Laxmi, 2008, Gathala et al., 2013, Mehla et al., 2000). If farmers with relatively smaller landholdings or otherwise lower socio-economic status systematically received services at a later time or with less attention to proper seed rate, fertilizer rate, and seed depth calibration, this would reduce the benefits of adoption. Based on data relating to the wheat growing season 2015, we explore quality determinants using a model of the form:

(4) $$y_{i}={\beta X}_{i}+\gamma S_{i}+{\varepsilon X}_{sp\left(i\right)}{+\theta {\mathrm{SP}}_{i}+v}_{i}$$

where $y_{i}$ measures ZT service quality on a scale of 1 (=very poor) to 5 (=excellent) according to the customer farmers’ perception. Due to an accumulation of observations at the lower and upper scale limits, we estimate a tobit model (Tobin, 1958) with heteroskedasticity-consistent standard errors that account for clustering of the sample at the village level. $X_{i}$ denotes a vector of customer (farm) characteristics, $S_{i}$ a vector of characteristics of the ZT service received by customer *i*, $X_{\mathrm{sp}(i)}$ is a vector of regressors related to the service provider, and ${\mathrm{SP}}_{i}$ is a measure of the density of ZT service providers around customer *i*; β,γ,ε and θ are vectors of parameters to be estimated, and $v_{i}$ is a random error term.

### 3.2 Model specifications

#### 3.2.1 ZT adoption

Based on Feder et al. (1985) and drawing on the concept of livelihood resources established in the sustainable livelihoods framework (Chambers and Conway, 1992, Scoones, 1998), we hypothesize the adoption of ZT in wheat to be determined by the households’ asset base and risk preferences. Table 1 provides the definitions and summary statistics of the dependent and explanatory variables used in the heckprobit models. It further shows that the first-stage equation contains two dummy explanatory variables, radio and TV ownership, which are omitted from the second-stage; with respect to information access, mass media channels are of particular importance for knowledge acquisition, whereas the adoption decision is influenced more by interpersonal communication channels (Rogers, 2003). Apart from being conceptually consistent, having at least one variable in the vector of selection equation regressors (*Z_i_*) which is not included in the regressors of the second stage (*X_i_*) is desirable for econometric reasons. If *Z_i_* and *X_i_* are identical, the IMR can be highly correlated with the elements of *X_i_*, leading to inflated standard errors (Wooldridge, 2006: 620). Conversely, *X_i_* should be a strict subset of *Z_i_*, i.e. all explanatory variables in the second-stage equation are included in the selection equation to ensure consistent estimates (ibid.).

**Table 1:** Definitions and summary statistics of dependent and explanatory variables in regression models explaining awareness and adoption conditional on awareness of zero-tillage (ZT) technology in wheat in Bihar (values are means, standard deviations in parentheses).

Variable description			2012/13		2015/16	
			Awareness (N = 923)	Adoption (N = 430)	Awareness (N = 923)	Adoption (N = 771)
*Dependent variables*						
ZT awareness	=	Dummy = 1 if HH^1^ head at least knows about ZT in theory, 0 otherwise	0.466 (0.499)	–	0.835 (0.371)	–
ZT adoption	=	Dummy = 1 if HH used ZT in wheat in the 2012/13 *rabi* season, 0 otherwise	–	0.693 (0.462)	–	0.481 (0.500)

*Natural capital*						
Cultivable area	=	Total area available for cultivation (ha)	1.254 (1.231)	1.675 (1.439)	1.331 (1.232)	1.440 (1.275)
Maximum plot size	=	Size of largest irrigable plot (ha)	0.595 (0.629)	0.757 (0.740)	0.628 (0.621)	0.682 (0.647)
Land owned	=	Dummy = 1 if HH head owns land, 0 otherwise	0.900 (0.300)	0.928 (0.259)	0.892 (0.311)	0.885 (0.320)

*Human capital*						
Labor/land ratio	=	Labor-to-land ratio (no. HH members aged 15 to 65 ha^−1^)	8.739 (14.520)	5.691 (8.366)	8.390 (15.200)	7.175 (11.762)
Age	=	Age of HH head (years)	49.573 (13.210)	49.042 (12.667)	52.806 (13.323)	52.668 (13.252)
High education	=	Dummy = 1 if educational achievement of HH head is > 12th grade, 0 otherwise	0.112 (0.315)	0.172 (0.378)	0.122 (0.328)	0.131 (0.338)
SC/ST category	=	Dummy = 1 if HH belongs to Scheduled Castes (SC) or Scheduled Tribes (ST), 0 otherwise^3^	0.122 (0.328)	0.102 (0.303)	0.122 (0.328)	0.112 (0.315)
General caste category	=	Dummy = 1 if HH belongs to one of the ‘general’ (non-marginalized) castes, 0 otherwise	0.407 (0.492)	0.472 (0.500)	0.407 (0.492)	0.419 (0.494)
Risk preference	=	HH head’s general risk preference, self-assessed on a scale from 0 (=fully avoiding risk) to 10 (=fully prepared to take risk)	5.167 (2.193)	5.674 (2.273)	5.988 (1.683)	6.005 (1.635)

*Financial capital*						
Credit access	=	Logged max. amount HH could currently borrow (‘000 INR)^2^	115.271 (244.925)	143.772 (259.640)	201.407 (234.049)	204.193 (245.555)

*Access to information and ZT services*						
Farmer association	=	Dummy = 1 if HH head is member of the local farmer association	0.021 (0.142)	0.026 (0.158)	0.036 (0.186)	0.042 (0.196)
Extension access	=	Access to agricultural extension on a scale from 0 (= no access) to 5 (= very good access)	2.619 (1.386)	2.840 (1.410)	2.619 (1.386)	2.677 (1.390)
Mobile phone	=	Dummy = 1 if HH owns at least one mobile phone, 0 otherwise	0.940 (0.237)	0.963 (0.189)	0.973 (0.162)	0.975 (0.155)
Radio	=	Dummy = 1 if HH owns at least one radio, 0 otherwise	0.244 (0.430)	–	0.102 (0.303)	–
TV	=	Dummy = 1 if HH owns at least one TV set, 0 otherwise	0.237 (0.426)	–	0.431 (0.496)	–
No ZT SP in 5 km	=	Dummy = 1 if there is no ZT service provider in 5 km radius, 0 otherwise	0.022 (0.146)	0.021 (0.143)	0.022 (0.146)	0.019 (0.138)

*Social network characteristics*						
NM ZT use*smallest	=	NM ZT use, interacted with smallest farm size tercile dummy variable	7.539 (23.763)	8.493 (25.571)	15.917 (35.039)	16.750 (35.864)
NM ZT use*middle	=	NM ZT use, interacted with middle farm size tercile dummy variable	13.100 (31.554)	16.172 (34.733)	14.910 (33.123)	17.417 (35.281)
NM ZT use*largest	=	NM ZT use, interacted with largest farm size tercile dummy variable	15.132 (33.483)	23.682 (39.657)	16.992 (34.904)	19.909 (37.058)
NM meet frequency	=	Average monthly number of contacts with NMs	9.225 (6.507)	9.413 (6.440)	10.295 (7.038)	9.902 (7.056)
NM age	=	Average age of NMs (years)	48.418 (8.275)	48.209 (7.831)	49.715 (8.123)	49.454 (8.056)
NM education	=	Total number of years of education of NMs	21.319 (12.119)	24.789 (12.441)	25.278 (11.521)	25.957 (11.387)
NM higher caste	=	Dummy = 1 if all NMs belong to a higher caste than the respondent, 0 otherwise	0.042 (0.201)	0.044 (0.206)	0.144 (0.351)	0.148 (0.355)

*District dummies (Bhojpur is base district)*						
Begusarai	=	Dummy = 1 if HH is located in Begusarai district, 0 otherwise	0.077 (0.267)	0.063 (0.243)	0.077 (0.267)	0.061 (0.239)
Bhojpur	=	Dummy = 1 if HH is located in Bhojpur district, 0 otherwise	0.481 (0.500)	0.558 (0.497)	0.481 (0.500)	0.519 (0.500)
Buxar	=	Dummy = 1 if HH is located in Buxar district, 0 otherwise	0.131 (0.338)	0.114 (0.318)	0.131 (0.338)	0.136 (0.343)
Lakhisarai	=	Dummy = 1 if HH is located in Lakhisarai district, 0 otherwise	0.076 (0.265)	0.081 (0.274)	0.076 (0.265)	0.088 (0.284)
Samastipur	=	Dummy = 1 if HH is located in Samastipur district, 0 otherwise	0.157 (0.364)	0.112 (0.315)	0.157 (0.364)	0.147 (0.354)
Vaishali	=	Dummy = 1 if HH is located in Vaishali district, 0 otherwise	0.078 (0.268)	0.072 (0.259)	0.078 (0.268)	0.049 (0.217)

1 HH = Household.

2 For ease of interpretation, summary statistics are provided for the unlogged variable.

3 Scheduled Castes and Scheduled Tribes are government recognized categories of marginalized groups that have historically been discriminated against and are both socially and economically disadvantaged. The Indian Constitution has provisions to protect their rights and provide them with equal opportunities. The base category which HHs belonging to the *SC/ST category* and the *General caste category* are evaluated against are those belonging to the ‘Other Backward Castes’ (OBC), constituting an intermediate social stratum.

Drawing on Matuschke and Qaim (2009), a salient feature of our model is the inclusion of the respondents’ individual agricultural information network characteristics as explanatory variables in both the awareness and adoption stages of our model. To this end, survey respondents were asked to provide some basic information on three farmers whom they interacted with most frequently about agricultural issues, termed network members (NMs) in our analysis. To capture endogenous network effects, we collected data on the NMs’ ZT adoption status, including the information whether the NM had adopted before or after the respondent farmer. The latter piece of information is crucial to address what Manski (1993) coined the reflection problem: while the behavior of the group (i.e., NMs) potentially influences the individual (i.e., the respondent), the reverse is also true. Hence, only those NMs who used ZT earlier than the respondent enter our model as ZT adopters, whereas those who adopted in the same year or later are considered non-adopters to avoid the reflection problem. To capture potential exogenous network effects, i.e., those caused by who the NMs are rather than how they behave, we include variables related to NMs’ age, education, and caste. To some extent, the inclusion of information on NM characteristics may mitigate the econometric problem that peer group membership itself is likely to be endogenous (Matuschke and Qaim, 2009, Songsermsawas et al., 2016) since individual social networks tend to be characterized by a high degree of homophily, i.e., they are usually formed among farmers of a similar social status (Rogers, 2003). A methodologically preferable approach would be the use of instrumental variables to control for potential endogeneity. Songsermsawas et al. (2016) used the characteristics of friends of the respondents’ network peers (who are unknown to the respondents themselves) as instruments for the peers’ characteristics, but such costly-to-collect data were not available in our case. As farmers of different socioeconomic status are likely to have differential access to formal information sources about agricultural innovations (see section 4.2 below), the information gained through personal communication with their self-reported peers may influence their adoption decisions to varying extents; we therefore disaggregate our estimated endogenous network effects by farm size tercile.

Since farmers’ access to the ZT technology largely depends on service providers (Erenstein and Laxmi, 2008, Keil et al., 2016), a variable reflecting the density of ZT SPs around the respondent household is included in the model. Using the GPS coordinates of ZT SPs in the survey districts, as monitored by CSISA, we created variables measuring the number of ZT SPs within various radiuses around individual sample households, ranging from 500 m to 20 km. Based on the explanatory power of the different variables tested, a dummy variable indicating the absence of any ZT SP in a 5 km radius enters the final model. Furthermore, district dummies control for location-specific differences, which may be caused by factors such as the varying timings and intensities of CSISA activities. The full set of explanatory variables is also used for the estimation of the multinomial logit model.

#### 3.2.2 ZT service quality

We hypothesize the rating of ZT service quality to be influenced by objectively measurable characteristics of the ZT service received, as well as by customer characteristics, ZT SP characteristics, and the density of SPs to reflect competition; furthermore, location-specific factors are controlled for. Based on data from a survey of ZT SPs in the area, we are able to match customer farmer responses with SP characteristics in 48 cases. Due to the small sample we estimate two models, where Model 1 omits and Model 2 includes SP characteristics. The definitions of the variables are straightforward as provided in Table 2. The variable *Training received* refers to training on proper seed and fertilizer rate calibration and other technical issues regarding operation of the ZT drill. We use two variables to reflect the level of competition among ZT SPs; in Model 1, the variable *SP density – Cust* measures the number of ZT SPs operating within a 5 km radius around the respondent household. In Model 2, we use the number of ZT SPs operating within a 5 km radius around the SP who served the respondent household (*SP density – SP*) as a superior proxy of the level of competition among service providers.

**Table 2:** Definitions and summary statistics of dependent and explanatory variables in Tobit models explaining the perceived quality of zero-tillage (ZT) services in Bihar (values are means, standard deviations in parentheses; SP = service provider).

			Model 1: without SP characteristics (N = 376)	Model 2: with SP characteristics (N = 48)
*Dependent variable*				
ZT service quality	=	Customer’s rating of ZT service quality on a scale of 1 (=very poor) to 5 (=excellent)	3.895 (0.920)	4.438 (0.649)

*ZT service characteristics*				
ZT service fee	=	ZT service fee per acre (INR)	824.069 (164.395)	715.625 (176.598)
ZT service delay	=	Number of days ZT service was delayed beyond desired time	1.144 (2.093)	0.396 (0.707)

*Customer characteristics*				
Cultivable area	=	Total area available to the customer for cultivation (ha)	1.543 (1.327)	1.507 (1.737)
Maximum plot size	=	Size of largest irrigable plot (ha)	0.821 (0.791)	0.917 (1.363)
High education	=	Dummy = 1 if educational achievement of service provider is > 12th grade, 0 otherwise	0.441 (0.497)	0.438 (0.501)
SP distance	=	Distance between customer’s and service provider’s homesteads (km)	0.776 (1.093)	0.629 (0.428)

*ZT service provider characteristics*				
SP cultivable area	=	Total area available to the service provider for cultivation (ha)	–	4.811 (2.828)
SP high education	=	Dummy = 1 if educational achievement of service provider is > 12th grade, 0 otherwise	–	0.354 (0.483)
Training received	=	Dummy = 1 if service provider received technical training on how to handle ZT drill, 0 otherwise	–	0.375 (0.489)
No training required	=	Dummy = 1 if service provider stated that technical training on how to handle ZT drill was not required, 0 otherwise	–	0.104 (0.309)
Years of ZT service provision	=	Number of years the service provider has provided ZT services	–	4.333 (0.519)
ZT service area	=	Total area covered with ZT services in 2016 (ha)	–	14.402 (8.541)

*ZT service provider density*				
SP density – Cust	=	Number of ZT service providers operating within a 5 km radius around the customer household	33.694 (18.921)	–
SP density – SP	=	Number of other ZT service providers operating within a 5 km radius around the service provider	–	31.824 (21.129)

*District dummies (Bhojpur is base district)*				
Begusarai	=	Dummy = 1 if HH is located in Begusarai district, 0 otherwise	0.051 (0.219)	0.333 (0.476)
Bhojpur	=	Dummy = 1 if HH is located in Bhojpur district, 0 otherwise	0.527 (0.500)	0.313 (0.468)
Buxar	=	Dummy = 1 if HH is located in Buxar district, 0 otherwise	0.223 (0.417)	0.000 (0.000)
Lakhisarai	=	Dummy = 1 if HH is located in Lakhisarai district, 0 otherwise	0.176 (0.381)	0.333 (0.476)
Samastipur	=	Dummy = 1 if HH is located in Samastipur district, 0 otherwise	0.011 (0.103)	0.000 (0.000)
Vaishali	=	Dummy = 1 if HH is located in Vaishali district, 0 otherwise	0.013 (0.115)	0.021 (0.144)

## 4 Results and discussion

### 4.1 Dynamics of awareness and use of zero-tillage wheat over time

The analysis of the 2013 survey data identified a significant scale bias in both ZT awareness and use of ZT technology. Among smaller-scale farmers, who were less educated and more likely to belong to lower castes, fewer knew about ZT and adopted the technology (Keil et al., 2017). Our data collected in 2016 from the same sample households shed light on whether this scale bias has persisted over the past three years. A comparison of farm sizes of ZT users versus non-users gives a first indication: in the 2012/13 *rabi* season, at 1.85 ha, the average farm of ZT users was approximately 73% larger than that of non-users at 1.07 ha (Mann-Whitney test significant at *P* < 0.001). Among farmers who started using ZT more recently, the difference in mean farm size compared to non-users amounted to only 18% (1.26 ha versus 1.07 ha; Mann-Whitney test significant at *P* < 0.01). To further examine the development of the social inclusiveness of ZT technology over time, in Table 3 we present information on basic farm characteristics and ZT awareness and use, differentiated by farm size terciles based on 2013 landholding information.

**Table 3:** Basic farm characteristics, ZT awareness, and use of ZT among sample households (HHs) in the 2012/13 and 2015/16 *rabi* seasons, differentiated by landholding tercile (based on 2013 operational holding information).

					2012/13		2015/16				
Farm size tercile	(1) Mean cultivable area (ha)^1^	(2) Mean size of largest irrigable plot (ha)^1^	(3) % HH heads with education <5th grade^2^	(4) % HH heads belonging to Scheduled castes^2^	(5a) % HHs knowing how ZT works^2^	(6a) % HHs using ZT^2^	(5b) % HHs knowing how ZT works^2^	(6b) % HHs using ZT^2^	(7) Increase in awareness rate rel. to 2013 (%)	(8) Increase in use rate rel. to 2013 (%)	(9) Stopped using ZT since 2013 (% of ZT users)^2^
Smallest (N = 323)	0.30^a^	0.21^a^	41.5	21.7	29.4	21.1	73.4	34.4	149.7	63.0	11.8 (N = 68)
Middle (N = 321)	0.90^b^	0.47^b^	25.9	11.2	44.5	30.8	84.4	38.0	89.7	23.4	15.2 (N = 99)
Largest (N = 317)	2.80^c^	1.24^c^	15.8	4.1	67.5	45.4	92.7	48.6	37.3	7.0	7.6 (N = 144)

Stat. sig.	^****^	^****^	^****^	^****^	^****^	^****^	^****^	^***^			n.s.

Whole sample (N = 961)	**1.32**	**0.64**	**27.8**	**12.4**	**47.0**	**32.4**	**83.5**	**40.3**	**77.7**	**24.4**	**10.9** (N = 311)

^*^(^**^){^***^}[^****^] Statistically significant at the 10% (5%) {1%} [0.1%] level of alpha error probability

1 Based on multiple Mann-Whitney tests, accounting for family-wise error.

2 Based on Chi-square test.

Columns 1–4 present basic farm and household head characteristics, illustrating the stark differences between groups in terms of farm size, size of the largest irrigable plot (which may be a limiting factor for the use of ZT in wheat), educational attainment and caste membership. Column 2 illustrates that plot size is much more likely to become a technically limiting factor for the smallest farmers and a factor that increases per-hectare transaction costs of ZT service provision, rendering very small farmers less attractive for service providers.

Looking at the development of awareness and use of ZT overall, Columns 5a and 5b show that the share of ZT-aware households rose from 47.0% in 2013 to 83.5% in 2016. Over the same period, the use rate of the technology increased from 32.4% to 40.3% (Columns 6a and 6b, respectively), commensurate to an increase of 24.4% (Column 7). By 2016, a total of 34 farmers (10.9%) who had used ZT in 2013 explicitly stated they had stopped using the practice (Column 9).

Differentiating our findings by farm size terciles reveals that lower-tercile farmers had a significantly lower rate of ZT awareness and ZT use compared to larger terciles in 2013 (Columns 5a and 6a). In 2016, we still observed highly significant differences between these groups (Columns 5b and 6b), but the gap between landholding terciles had narrowed: both with respect to ZT awareness and ZT use, the growth rate was higher for farmers in the lower terciles (Columns 7 and 8).

Overall, the share of ZT-aware farmers who were using the technology amounted to 68.9% in 2013, with very little variation across landholding terciles. Since then, awareness of ZT has grown faster than the use of the technology, lowering the proportion of ZT users among the knowledgeable sub-group to 48.3% in 2016. The share among the largest landholding tercile was slightly higher (52.4%) than that among the middle and smallest terciles (45.0% and 46.9%, respectively).

### 4.2 Dynamics of farmers’ individual agricultural information networks

Among the respondents who knew about ZT in 2013, 73.1% cited ‘fellow farmers’ as their primary source of information about the technology, indicating that farmers’ individual information networks may play an important role in the diffusion of ZT technology. Furthermore, the share of respondents citing other farmers as primary information source differed significantly between farm size terciles, amounting to 90.0%, 71.8%, and 66.7% among the smallest, middle, and largest tercile respondents, respectively (chi-square test significant at *P* < 0.001). Hence, social network effects may differ between socioeconomic strata, warranting the estimation of disaggregated coefficients in the subsequent regression analysis.

Table 4 shows basic characteristics of our respondents’ individual social networks at the times of data collection in 2013 and 2016, differentiating by farm size terciles and ZT adoption status. Column 1 shows that also the farm sizes of the respondents’ NMs differed significantly across landholding terciles, illustrating that farmers tended to interact with other farmers of similar landholding size, with minor ‘upward orientation’ in self-reported peers apparent for respondents in the smallest and middle terciles. Consistent with our finding that ZT adopters had larger farms than non-adopters (see Section 4.1), also the mean farm size of adopters’ NMs exceeded that of non-adopters’ NMs.

**Table 4:** Major characteristics of respondents’ personal network members^1^ (NMs), differentiated by respondents’ farm size tercile^2^ and zero-tillage (ZT) adoption status (values are means based on 2013 and 2016 survey rounds).

Respondent characteristics	(1) Farm size of NMs (ha)^2^		(2) Years of Education of NMs		(3) No. of NMs belonging to Scheduled castes		(4) No. of ZT users among NMs		(5) No. of NM ZT users who had adopted earlier than respondent	
	2013	2016	2013	2016	2013	2016	2013	2016	2013	2016
*Farm size tercile*										
Smallest (N = 323)	1.43^a^	1.48^a^	6.19^a^	7.97^a^	0.45^a^	0.38^a^	0.73^a^	1.58	0.61^a^	1.49
Middle (N = 321)	1.94^b^	1.56^b^	7.77^b^	8.35^a^	0.25^b^	0.25^b^	1.20^b^	1.48	1.04^b^	1.35
Largest (N = 317)	3.07^c^	2.10^c^	8.92^c^	9.06^c^	0.09^c^	0.07^c^	1.67^c^	1.60	1.28^c^	1.41
Stat. sig.	^****^	^**^	^****^	^**^	^***^	^*^	^****^	n.s.	^**^	n.s.

Whole sample (N = 961)	2.16	1.71	7.62	8.45	0.26	0.23	1.20	1.56	0.97	1.42

*ZT adoption status*^3^										
Adopters (N = 311/369)	2.89	2.18	8.89	9.14	0.19	0.19	1.99	2.43	1.33	2.15
Non-adopters (N = 642/433)	1.81	1.34	7.00	7.82	0.30	0.29	0.81	0.77	0.81	0.76
Stat. sig.	^****^	^****^	^****^	^****^	^***^	^*^	^****^	^****^	^****^	^****^

Whole sample (N = 953/802)	2.16	1.72	7.61	8.43	0.27	0.24	1.20	1.53	0.98	1.40

*(**){***}[****] Statistically significant at the 10% (5%) {1%} [0.1%] level of alpha error probability, based on (multiple) Mann-Whitney tests, accounting for family-wise error; diverging superscript letters indicate statistical significance *at least* at the indicated level.

1 Those three farmers whom the respondent communicated with most about agricultural issues.

2 Based on 2013 landholding information.

3 For a clear differentiation, disadopters were excluded from this analysis, reducing sample size to 953 observations in 2013 and 802 observations in 2016.

With respect to educational achievement and caste membership, Columns 2 and 3 reveal similar patterns among NMs as among the survey respondents themselves (cf. Table 3, Columns 3 and 4), corroborating our finding that farmers interacted mostly with farmers who were close to their own social stratum, i.e. they tended to form homophilous social networks (Rogers, 2003). As would be expected, these patterns did not change significantly between 2013 and 2016.

However, the comparison of ZT users among NMs of different landholding terciles exhibits significant changes over time (Columns 4 and 5): in 2013, respondent farmers in the largest landholding tercile had more than twice as many ZT users among their NMs as farmers in the smallest tercile, with farmers in the middle tercile taking an intermediate position (all differences statistically significant). In 2016, there was no significant difference in the number of ZT users among NMs across farm size terciles, which is consistent with our previous finding that the use of ZT had increased more than proportionately among smaller-scale farmers (Table 3). For our regression analysis Column 5 is of particular relevance, indicating the number of ZT users among NMs who had adopted the technology prior to the survey respondents, hence potentially influencing the respondents’ knowledge and use of the practice.

The relevance of social networks in the adoption process is corroborated by the comparison between ZT adopters and non-adopters in the lower part of Table 4, illustrating that survey respondents who had adopted ZT had more ZT users among their NMs than those who had not adopted the practice. While the difference is statistically highly significant in both observed years, the gap was wider in 2016 than in 2013 (Columns 4 and 5).

### 4.3 Dynamics of factors affecting knowledge and adoption of zero-tillage wheat

#### 4.3.1 Estimates of the heckprobit models

To test whether the estimation of separate regressions for *rabi* seasons 2012/13 and 2015/16 is justified, we estimated a pooled regression including time-interaction terms for each explanatory variable and used a likelihood-ratio test to test their joint significance as compared to a pooled regression containing a season dummy variable only. The test strongly rejected the null-hypothesis, justifying the estimation of separate regressions^7^. For illustrative purposes, the pooled regression containing a season dummy is presented in the Annex (Table A1).

Table 5 presents the estimates produced by the separate heckprobit models explaining knowledge of ZT and, conditional on being knowledgeable, use of ZT wheat in 2012 and 2015 (Models 1 and 2, respectively). The models were tested for potential multicollinearity, and no cause for concern was found. Their explanatory power in terms of overall share of cases correctly predicted amounts to 72.3% in Model 1 and 83.0% in Model 2 (bottom row of Table 5). While both models produce predicted adoption probabilities that differ highly significantly between observed adopters and observed non-adopters, Model 2 is by far superior in correctly predicting both cases of adoption and non-adoption,^8^ which may be due to the substantially larger sample in the second-stage estimation as well as the larger number of adopters.

**Table 5:** Maximum Likelihood estimates of Heckman probit selection models explaining awareness of zero-tillage (ZT) and adoption of ZT wheat conditional on awareness in *rabi* seasons 2012/13 and 2015/16 in Bihar; coefficients are marginal effects.

	Model 1: *Rabi* season 2012/13				Model 2: *Rabi* season 2015/16			
	Awareness		Adoption cond. on awareness		Awareness		Adoption cond. on awareness	
Variable	Coefficient^1^	z-value^2^	Coefficient^1^	z-value^2^	Coefficient^1^	z-value^2^	Coefficient^1^	z-value^2^
Cultivable area	0.1478	3.44^***^	0.1076	2.65^***^	0.0735	1.81^*^	0.0618	1.39
Cultivable area, sqd.	−0.0118	−1.78^*^	−0.0136	−2.11^**^	−0.0075	−1.31	−0.0117	−2.16^**^
Maximum plot size	−0.0053	−0.13	0.0423	1.17	0.0016	0.04	0.0617	1.97^**^
Land owned^d^	0.0510	1.06	0.1321	2.26^**^	−0.0404	−0.94	−0.0482	−1.88^*^
Labor/land ratio	−0.0041	−2.35^**^	−0.0022	−1.37	−0.0011	−2.32^**^	−0.0018	−1.55
Age	−0.0009	−0.70	−0.0009	−0.68	−0.0010	−1.11	0.0002	0.22
High education^d^	0.0903	1.84^*^	0.0627	1.12	−0.0067	−0.24	0.0752	2.07^**^
SC/ST category^d^	0.0397	0.71	−0.0163	−0.25	−0.0759	−2.56^**^	−0.0166	−0.39
General caste category^d^	0.0647	1.72^*^	0.0871	1.92^*^	0.0011	0.04	0.0860	2.00^**^
Risk preference	0.0212	3.12^***^	0.0264	4.05^****^	0.0310	3.36^***^	0.0180	1.39
Credit access	0.0008	0.24	−0.0040	−1.33	0.0074	0.52	0.0133	0.83
Farmer association^d^	−0.0050	−0.04	0.1260	1.18	0.0769	0.86	0.0820	1.22
Extension access	0.0273	2.53^**^	−0.0002	−0.02	0.0079	1.15	−0.0102	−1.10
Mobile phone^d^	−0.0076	−0.13	−0.0158	−0.19	−0.0348	−0.47	0.0217	0.27
Radio^d^	0.0999	4.05^****^	–		0.0028	0.08	–	
TV^d^	−0.0204	−1.31	–		0.0602	2.17^**^	–	
No ZT SP in 5 km^d^	0.1145	2.46^**^	−1.3411	−8.35^****^	−0.0480	−1.27	−0.6014	−3.43^***^
NM ZT use*smallest	0.0027	4.57^****^	0.0019	3.07^***^	0.0020	4.32^****^	0.0031	5.39^****^
NM ZT use*middle	0.0018	2.93^***^	0.0010	1.69^*^	0.0029	5.73^****^	0.0033	8.44^****^
NM ZT use*largest	0.0009	1.62	0.0006	1.28	0.0017	2.43^**^	0.0024	4.60^****^
NM meet frequency	0.0080	3.11^***^	0.0056	1.74^*^	−0.0001	−0.04	−0.0008	−0.25
NM age	−0.0015	−0.97	−0.0021	−1.12	−0.0023	−2.05^**^	0.0011	0.57
NM education	0.0046	3.70^****^	0.0028	2.16^**^	0.0012	1.13	−0.0002	−0.11
NM higher caste^d^	0.0266	0.50	0.0816	1.35	0.0028	0.06	0.0503	1.07
Begusarai^d^	−0.2192	−5.56^****^	−0.1214	−2.41^**^	−0.2523	−5.09^****^	−0.0758	−1.48
Buxar^d^	−0.0845	−1.43	0.0147	0.25	−0.1616	−4.36^****^	0.1957	3.24^***^
Lakhisarai^d^	−0.1869	−4.45^****^	−0.1412	−4.41^****^	0.0556	0.82	0.3863	5.71^****^
Samastipur^d^	−0.1760	−3.37^***^	−0.2863	−2.95^***^	−0.1408	−2.33^**^	−0.4077	−3.48^****^
Vaishali^d^	−0.1383	−2.00^**^	−0.2717	−3.03^***^	−0.3486	−9.02^****^	−0.4818	−8.00^****^

N =	923		430		923		771	
Log pseudolikelihood =			−705.90				−587.08	
Wald test of independent equations: chi-square (1) =			6209.60^****^				0.04	

*Explanatory power:*								
Cases of ZT adopters correctly predicted (%) =			39.3				80.9	
Cases of ZT non-adopters correctly predicted (%) =			88.0				83.0	
Overall cases correctly predicted (%) =			72.3				82.1	

^*^(^**^)[^***^]{^****^} Significant at the 10%(5%)[1%]{0.1%} level of alpha error probability.

1 Coefficients are marginal effects (evaluated at means of all explanatory variables); for dummy variables, marginal effects are for a discrete change from 0 to 1.

2 Based on robust standard errors adjusted for 40 village-level clusters.

d Dummy variable.

In the following, we highlight the key findings and differences between Models 1 and 2. The variable *Cultivable area* is included in the models as a wealth indicator and a factor that may influence the adoption of ZT directly, as the provision of ZT services on small farms is associated with higher per-hectare transaction costs and, hence, is likely less attractive for service providers (Keil et al., 2016). Model 1 indicates a statistically significant positive quadratic relationship both with respect to awareness of ZT (henceforth awareness stage) and adoption of the technology conditional on being aware (henceforth adoption stage). The magnitude of the coefficients indicates that the marginal effect would turn negative only beyond a farm size of 12.5 ha^9^ and 7.9 ha in the awareness and adoption stages, respectively. Given an average farm size of 1.3 ha and a 99% percentile of 6.5 ha, this means that the marginal effect of farm size remained positive across the entire range of landholdings usually encountered in the research area. Hence, in 2012, there was a clear bias in favor of larger-scale farmers, but the bias decreased at the margin, i.e. it was most pronounced among the smallest landholders. In contrast, there was no clear influence of farm size in 2015^10^. In addition to farm size, plot size may influence the adoption of ZT. Very small plots may pose a technical limit to operating four-wheel tractor-based equipment; moreover, per-hectare transaction costs for ZT services increase with decreasing plot size, potentially influencing SPs’ willingness to service very small plots. We therefore included the variable *Max. plot size* in the models, which measures the size of the largest irrigable plot available.^11^ We find no significant effect of *Max. plot size* in 2012, but a significant and positive effect in the adoption stage in 2015. This indicates that, while the share of ZT users among smaller-scale farmers increased over time, farmers with less fragmented land were more likely to use the technology, possibly because it was easier for them to avail custom-hiring services. While there is strong evidence of immediate benefits from the use of ZT in wheat in Bihar in terms of yield increase and cost savings (Keil et al., 2015), *Land owned* was included as a control variable for land tenure status. While land owners were more likely to use ZT in 2012, the weakly significant negative coefficient in the adoption stage in 2015 indicates that farmers are also willing to use it on rented land, which is likely related to their growing confidence in the technology’s short-term benefits.

Regarding human capital, several factors are significant in the awareness and adoption stages. The labor/land ratio has a negative effect on the awareness of ZT in both models, indicating that households with relatively abundant family labor are less likely to gather information about novel mechanized technologies, which are generally labor-saving. Finding no significant effect on ZT adoption is plausible as the alternative to hiring ZT services is usually the hiring of plowing services, affecting hired labor rather than family labor use. *High education* increased the likelihood of being aware of ZT by approx. 9.0 percentage points in 2012 and the likelihood of adopting (conditional on awareness) by approx. 7.5 percentage points in 2015. The partly significant coefficients on *SC/ST category* and *General caste category* indicate some influence of caste membership (in the expected direction) in both years. Based on our self-assessment measure of *Risk preference*, we find strong evidence that less risk-averse farmers were significantly more likely to be informed about ZT in both years. While less risk-averse farmers were more likely to use the technology in 2012, the data do not support any significant effect in the adoption stage in 2015. Due to its proven short- and long-term benefits (cf. Mehla et al., 2000, Erenstein and Laxmi, 2008, Chauhan et al., 2012, Gathala et al., 2013, Keil et al., 2015), the use of ZT in wheat is objectively a risk-reducing technology. However, in 2011 only 134 ZT SPs operated in CSISA’s focal geographies in Bihar, catering to a total of 2095 customers and covering a total of 1720 ha. Hence, at the time of the initial survey, ZT was an unfamiliar technology to most farmers; as with other agricultural innovations at an early stage of diffusion, ZT was likely *perceived* to be risky at the time (Feder et al., 1985, Rogers, 2003 f.). Our finding indicates that, as the experience with the practice grows and it becomes more widespread, this perception is overcome.

Access to agricultural extension enhanced awareness of ZT in 2012, but the data do not support a significant effect in 2015. In both years mass media had a significant effect. Interestingly, in 2012, radio was the relevant medium while in 2015 it was television; TV ownership increased from 24% to 43% of survey households over the same time (see Table 1).

The variables related to farmers’ informal social networks yield a number of highly significant coefficients: in 2012 the NMs’ adoption of ZT positively influenced the awareness and use of the technology by farmers in the smallest and middle landholding terciles (NM ZT use*smallest and NM ZT use*middle); a one-percentage point increase in the ZT adoption rate among the smallest (middle) farmers’ NMs entailed a 0.19 (0.10) percentage point increase in their own propensity to adopt the technology. For the largest-tercile farmers, the estimated coefficient is also positive, but not significantly different from zero. Model 2 shows that these endogenous individual network effects were even more pronounced in 2015: both in the awareness and adoption stages the magnitude of coefficients and their significance levels are generally larger than in Model 1, and the effects are statistically significant also for farmers in the largest landholding tercile. Apart from these highly significant endogenous network effects, we find that the NMs’ level of education (*NM education*) positively affected both awareness and use of ZT in 2012, representing an exogenous network effect. The latter was not observed in 2015, i.e. how the NMs behaved was much more relevant than how educated they were.

Since for most farmers the use of ZT hinges on access to custom-hiring services, the likelihood of using the technology declined drastically for households with no ZT SP within a 5 km radius. This emanates from both models, whereby the magnitude of the effect was larger in 2012 than in 2015.^12^ The positive and significant coefficient in the awareness stage in Model 1 is counter-intuitive, but it illustrates that farmers’ knowledge of the technology did not hinge on the presence of a local ZT SP.

Finally, dummy variables control for systematic differences between districts. ZT related CSISA activities started in Bhojpur district, which serves as the base district in the models. In 2012, all statistically significant coefficients are negative, which is likely related to the shorter time of exposure to ZT technology and the lag in the development of the respective service economy. In the 2015 model, it is interesting to note that the coefficient on the district *Lakhisarai* has turned positive and very substantial in magnitude (0.39) while the coefficients on *Samastipur* and *Vaishali* have grown more negative. This illustrates that, geographically, we observe a divergence in the uptake of ZT technology rather than a convergence. Primary causes for this divergence are likely related to differences in topography and the prevailing cropping systems between districts, ultimately affecting soil moisture and weed pressure: ZT conserves soil moisture and typically enables farmers to forego pre-sowing irrigation for wheat, hence conserving water resources and reducing irrigation costs (Erenstein & Laxmi, 2008). However, under conditions of relatively light soils and slightly elevated topography, as found especially in Begusarai, Samastipur and Vaishali districts, the soil may be too dry at the time of wheat sowing, requiring farmers to apply pre-sowing irrigation after prior soil tillage. In addition, the use of short-duration hybrid rice is increasing in these districts, leading to earlier harvesting (in the first half of October) and a prolonged period of uncovered soil before wheat can be sown in early November. Again, the resulting evaporative losses of soil moisture during this period may make pre-sowing irrigation necessary. Moreover, weeds may emerge in the intervening period, which many farmers prefer to control through conventional tillage (R. K. Malik, CSISA agronomist and Bihar operations manager, personal communication).

#### 4.3.2 Estimates of the multinomial logit model

Out of the 923 panel data observations that enter the heckprobit estimation, 894 (96.9%) could be clearly classified as falling into one of the four distinct adoption categories. The number of ‘Early adopters’ amounted to 182 (20.4%) and the number of ‘Recent adopters’ to 181 (20.3%), 424 respondents (47.4%) were ‘Non-adopters’, and 107 respondents (12.0%) fell into the ‘Dis-adopter’ category. Among the ‘Early adopters’, 72.5% had started using ZT in 2010, and 8.2% and 19.2% had started in 2011 and 2012, respectively. Ninety-five percent of the ‘Recent adopters’ had started the practice in 2013 and the remainder in 2014 (2.8%) and 2015 (2.2%).

We confirmed the appropriateness of the multinomial logit model for our application by testing the Independence of Irrelevant Alternatives (IIA) assumption using the Hausman-McFadden test (Hausman & McFadden, 1984). In addition, a likelihood-ratio test rejected the null-hypothesis that any two of the outcome categories can be combined. Using the full set of explanatory variables that were applied in the heckprobit estimation,^13^ Table 6 presents the marginal effects based on the multinomial logit model. The coefficients indicate the absolute effect of a one-unit change in the respective variable on the probability of each outcome, evaluated at the means of all explanatory variables.^14^ The findings corroborate the heckprobit results, but exhibit a somewhat clearer pattern as early adopters, recent adopters, non-adopters and dis-adopters are separated. Starting with the commonalities, the absence of a proximate service provider (*No ZT SP in 5 km*) has a statistically highly significant effect on the probability of falling into any one of the four groups. The marginal effect is negative and of similar magnitude for early adopters (−0.38) and recent adopters (−0.35). Likewise, it is negative and of great magnitude and statistical significance for the group of dis-adopters. While this may seem counter-intuitive, dis-adopters once belonged to the early ZT users and must typically have had access to a ZT SP before deciding to discontinue the practice. As to be expected, the absence of a proximate ZT SP has a very large positive effect on non-adoption of the technology. Another commonality concerns the relevance of endogenous social network effects: the ZT adoption behavior of their NMs has highly significant effects on early adopters, recent adopters, and non-adopters across all landholding terciles. The marginal effects on recent adopters are greater in magnitude than those on early adopters, especially for the largest landholding tercile. Network effects do not seem to be relevant for the group of dis-adopters; however, it must be kept in mind that our data did not capture potential discontinuation of ZT use among NMs.

**Table 6:** Maximum Likelihood estimates of a multinomial logit model explaining different categories of zero-tillage (ZT) wheat adoption in Bihar (definitions below the table); coefficients are absolute marginal effects evaluated at the means of all explanatory variables.

	Early adoption (N = 182)		Recent adoption (N = 181)		Non-adoption (N = 424)		Dis-adoption (N = 107)	
Variable	Coefficient^1^	z-value^2^	Coefficient^1^	z-value^2^	Coefficient^1^	z-value^2^	Coefficient^1^	z-value^2^
Cultivable area	0.1146	3.76^****^	−0.0595	−1.77^*^	−0.0206	−0.41	−0.0345	−0.96
Cultivable area, sqd.	−0.0112	−2.80^***^	−0.0013	−0.27	0.0059	0.85	0.0066	1.57
Maximum plot size	−0.0113	−0.49	0.0665	2.75^***^	−0.0381	−0.93	−0.0171	−0.58
Land owned^d^	−0.0528	−1.22	−0.0206	−0.60	0.0154	0.31	0.0581	1.85^*^
Labor/land ratio	−0.0032	−1.04	0.0016	0.91	0.0051	2.98^***^	−0.0035	−2.14^**^
Age	−0.0003	−0.37	−0.0005	−0.50	0.0012	1.19	−0.0004	−0.52
High education^d^	0.0858	2.74^***^	−0.0264	−0.74	−0.0658	−1.76^*^	0.0064	0.17
SC/ST category^d^	−0.0018	−0.04	−0.0388	−1.28	0.0918	2.39^**^	−0.0512	−1.76^*^
General caste category^d^	0.0565	1.76^*^	0.0171	0.60	−0.0506	−1.08	−0.0230	−0.66
Risk preference	0.0241	2.51^**^	0.0012	0.13	−0.0110	−1.07	−0.0143	−1.48

Credit access	0.0101	0.76	0.0159	1.21	−0.0084	−0.50	−0.0176	−1.46

Farmer association^d^	0.0847	1.26	0.0048	0.09	−0.1101	−1.39	0.0206	0.28
Extension access	0.0002	0.02	−0.0101	−1.01	−0.0005	−0.06	0.0105	1.33
Mobile phone^d^	0.0897	1.10	−0.0517	−0.99	0.0621	0.73	−0.1001	−1.05
Radio^d^	−0.0074	−0.15	0.0737	1.37	−0.0977	−1.96^*^	0.0314	0.72
TV^d^	−0.0006	−0.03	0.0157	0.64	−0.0363	−1.14	0.0213	0.76
No ZT SP in 5 km^d^	−0.3782	−2.54^**^	−0.3504	−2.89^***^	1.7090	6.61^****^	−0.9805	−7.11^****^

NM ZT use*smallest	0.0012	2.62^***^	0.0021	5.36^****^	−0.0029	−4.63^****^	−0.0004	−0.79
NM ZT use*middle	0.0017	5.10^****^	0.0021	7.42^****^	−0.0039	−8.69^****^	0.0002	0.46
NM ZT use*largest	0.0009	2.15^**^	0.0021	5.07^****^	−0.0032	−5.35^****^	0.0002	0.35
NM meet frequency	−0.0051	−2.43^**^	0.0047	2.20^**^	0.0010	0.39	−0.0006	−0.19
NM age	0.0011	0.69	0.0001	0.07	0.0002	0.13	−0.0014	−0.87
NM education	0.0020	1.05	−0.0011	−0.82	−0.0007	−0.45	−0.0001	−0.12
NM higher caste^d^	−0.0955	−3.20^***^	0.0914	1.64	0.0090	0.18	−0.0049	−0.11
Begusarai^d^	−0.0696	−1.75^*^	−0.0738	−1.45	0.0967	2.34^**^	0.0466	0.87
Buxar^d^	0.0855	2.21^**^	0.0040	0.10	−0.0706	−0.98	−0.0190	−0.28
Lakhisarai^d^	0.0855	2.67^***^	0.2422	4.60^****^	−0.2246	−2.62^***^	−0.1032	−1.73^*^
Samastipur^d^	−0.1911	−2.48^**^	−0.2900	−3.99^****^	0.4010	4.68^****^	0.0801	0.84
Vaishali^d^	−0.2233	−1.87^*^	−0.2954	−2.81^***^	0.4406	6.06^****^	0.0782	1.51

N =	894							
Log pseudolikelihood =	−760.32							
Pseudo R^2^ =	0.323							

^*^(^**^)[^***^]{^****^} Significant at the 10%(5%)[1%]{0.1%} level of alpha error probability.

^1^Coefficients are marginal effects (evaluated at means of all explanatory variables); for dummy variables, marginal effects are for a discrete change from 0 to 1.

^2^Based on robust standard errors adjusted for 40 village-level clusters.

^d^Dummy variable.

Definitions:

Early adoption = continuous use of ZT since 2012 or earlier (data available until 2010).

Recent adoption = continuous use of ZT since 2013 or later (data available until 2015).

Non-adoption = never used ZT throughout the period 2010–2015.

Dis-adoption = used ZT earlier, but did not use it for at least three consecutive years and/or stated that they stopped using ZT.

Common factors affecting non-adoption and dis-adoption, but in opposite directions, are *Labor/land ratio* and *SC/ST category*. While relative abundance of family labor favors non-adoption of ZT, the opposite is the case for dis-adoption; as indicated above, dis-adopters started using ZT at a relatively early stage, and relative labor scarcity may have been one motivating factor for testing a novel labor-saving technology. Along similar lines, early testers of ZT (and later dis-adopters) were less likely to belong to marginalized castes, as opposed to non-adopters (Table A3).

Since dis-adopters of ZT used to be (early) adopters of the technology, the potential of regression analysis to explain dis-adoption via differences in household characteristics is limited. We therefore asked those respondents who declared that they had discontinued the practice about their reasons to do so (N = 62). The primary reasons stated were (i) lacking access to timely ZT services (38.7%), (ii) lack of soil moisture necessitating pre-sowing irrigation with prior tillage (14.5%), (iii) reduced wheat yields due to increased weed infestation (12.9%), and (iv) high maintenance and repair costs of the ZT drill (11.3%). While reasons (i) and (iv) are self-explanatory, the implications of soil moisture and weed pressure variations for ZT adoption are elaborated in Section 4.3.1.

Of particular interest in this study is the comparison of factors affecting early adoption versus recent adoption. The model results clearly show that larger, better educated, and high-caste farmers were more likely to adopt ZT at an early stage. A risk-taking nature was another factor conducive to testing this novel technology at an early stage of diffusion. For recent adoption, farm size does *not* show up as a conducive factor any longer; in fact we estimate a weakly significant *negative* coefficient. Rather, plot size becomes a decisive factor, which was not the case for early adoption on larger landholdings.

To further elucidate (dis-)similarities between households in the four adoption categories, Table A3 in the Annex presents summary statistics for key variables, indicating statistically significant differences between groups. The descriptive statistics corroborate the regression results and also show significant differences between adopters and dis-adopters regarding access to ZT service providers and uptake of the technology among network members.

### 4.4 Factors affecting the quality of zero-tillage services

There is substantial variation in the rating that respondents assigned to the quality of ZT services received in 2015. Ranging from 1 to 5, the quality rating averaged 3.9, with 25%, 50% and 75% percentiles amounting to 3, 4, and 4.75, respectively. Table 7 presents the estimates of the tobit models explaining the quality of ZT services based on the respondents’ subjective assessment. Not accounting for SP characteristics, Model 1 uses all available cases of ZT users who had accessed the technology through service providers in 2015. The results indicate that a higher service fee was associated with better service quality. The timeliness of the service had a strong influence on the quality rating, with service delays resulting in a reduction of 0.19 points per day; the importance of timely wheat establishment to avoid terminal heat stress was emphasized in Section 3.1.4 above. Furthermore, a larger plot size was associated with a higher quality rating. Together with our heckprobit estimates for 2015 and the results of the multinomial logit model for ‘Recent adopters’, this indicates that land fragmentation influenced both the use of ZT and the quality of service received (while overall landholding size affected neither). The quality of ZT services also varied significantly across locations.

**Table 7:** Maximum Likelihood estimates of tobit models explaining the perceived quality of zero-tillage (ZT) services in Bihar (scale of 1 = very poor to 5 = excellent); coefficients are marginal effects.

	Model 1: without SP characteristics		Model 2: with SP characteristics	
Variable	Coefficient^1^	t-value^2^	Coefficient^1^	t-value^3^
ZT service fee	0.0021	4.22^****^	0.0002	0.29
ZT service delay	−0.1891	−6.29^****^	−0.3725	−1.99^*^
Cultivable area	−0.1201	−1.44	−0.7551	−1.60
Maximum plot size	0.3765	2.40^**^	1.4609	2.59^**^
High education^d^	0.0847	0.68	−0.4584	−1.37
SP distance	−0.0649	−0.91	0.7269	1.04

SP cultivable area	–		0.4166	3.95^****^
SP high education^d^	–		0.5573	2.64^**^
Training received^d^	–		1.0365	1.62
No training required^d^	–		2.3304	4.31^****^
Years of ZT service provision	–		0.8955	2.41^**^
ZT service area	–		−0.0399	−1.66

SP density – Cust	−0.0082	−1.54		
SP density – SP	–		0.0704	3.37^***^

Begusarai^d^	1.4740	4.42^****^	4.5454	7.02^****^
Buxar^d^	0.1402	0.68	–	
Lakhisarai^d^	−1.0281	−4.33^****^	1.1503	2.70^**^
Samastipur^d^	0.3629	1.75^*^	–	

Constant	2.7927	6.94^****^	−6.1009	−2.45^**^

N =	376		48	
No. left/right-censored obs. =	6/94		0/24	
Log pseudolikelihood =	−469.20		−36.08	
Pseudo R^2^ =	0.138		0.345	

^*^(^**^)[^***^]{^****^} Significant at the 10%(5%)[1%]{0.1%} level of alpha error probability.

1 Coefficients are marginal effects; for dummy variables, marginal effects are for a discrete change from 0 to 1.

2 Based on robust standard errors adjusted for 106 clusters of unique ZT service providers.

3 Based on robust standard errors adjusted for 15 clusters of unique ZT service providers.

d Dummy variable.

Model 2, which controls for SP characteristics, confirms the findings from Model 1 with respect to service delays and plot size. Despite the limited sample size, Model 2 indicates that SP characteristics also had an important bearing on the quality of service delivered: the SPs’ own farm size, educational achievement, and years of experience in ZT service provision all had a significant positive effect on the quality rating. Furthermore, the model suggests that technical training resulted in better-quality services, adding approximately one point to the quality rating as compared to SPs who had not received any training. This effect is statistically significant at *P* = 0.12 only, which may be attributable to the small sample size. Among the 30 sample SPs (62.5%) who had not received any training, there were five who claimed that they did not require any such training; indeed, their quality rating exceeded that of the remaining 25 SPs without training by 2.3 points. The model also suggests that there was a slight trade-off between the total area serviced and the quality of service provided (statistically significant at *P* = 0.11 only). Finally, the coefficient on the variable *SP density – SP* indicates that greater competition among SPs had a small – but statistically highly significant – quality enhancing effect.

## 5 Conclusions and recommendations

In this study, we investigated the dynamics of farmers’ use of ZT wheat in Bihar and explored how determinants of farmers’ knowledge and use of the technology changed over a three-year period. In 2012, when ZT was in its nascent stage of adoption in Bihar, better-educated and higher-caste farmers with larger landholdings were clearly more likely to know about and use ZT. Use rates among farmers in the largest landholding tercile exceeded that of farmers in the smallest tercile by 152%, corroborating the critique that it is mostly the better-off farmers who benefit from SI initiatives, as argued by Rasmussen et al. (2018). However, over the subsequent three-year period, awareness and use of the technology increased more than proportionately among less-educated farmers with smaller landholdings, narrowing the gap in ZT use rates between the largest and smallest terciles to 41%. Hence, the initial scale bias declined substantially over time. Education and caste did not significantly affect recent ZT adoption, and land *fragmentation* rather than total landholding size became a significant influencing factor.

Beyond the household level, physical proximity of ZT SPs remained an important determinant of ZT use among recent adopters as most farmers access the technology via custom-hiring services. For ZT SPs, apart from the total area serviced per customer, area fragmentation influences transaction costs and, hence, the relative attractiveness of a customer. Our analysis suggests that land fragmentation also affects the *quality* of ZT services, with poorer quality being delivered on smaller plots. In conclusion, farmers with small but contiguous landholdings may have adequate access to quality ZT services. Smallholders with fragmented landholdings, however, may be disadvantaged both with respect to access to ZT services in general, but also with respect to the quality of the service received. Poor quality – be it in terms of delayed service or poor machine calibration or both – may lead to adverse outcomes, such as terminal heat stress caused by delayed establishment or a poor crop stand due to improper machine calibration. Such experiences may easily discourage farmers from continuing to use the practice.

Our descriptive analysis of ZT use across social network members (Table 4) illustrates the growing social inclusiveness of the practice over time, and the econometric models corroborate the important role of networks in the adoption process. Network effects are even stronger with respect to recent than early adoption and apply to all landholding terciles. This is plausible as, with greater diffusion of the technology, there is greater scope for farmers to learn from existing ZT users’ experience. Of course, this means that also negative experiences due to poor-quality services will be easily shared and can have significant ripple effects, which highlights the importance of proper training of ZT SPs. Our analysis indicates significant variation in the quality of ZT services and suggests that technical training does enhance quality.

Furthermore, our analysis corroborates previous research that found social networks to be relatively homophilous, with limited social interaction across socioeconomic strata within a village. Hence, if agricultural extension messages are primarily diffused through ‘progressive’ farmers, who usually belong to the better-off, better-educated, and higher-caste stratum, they have limited scope of reaching the poorer segments in a village. To further boost farmers’ awareness of the technology, extension messages should therefore be targeted to farmers representing different social strata.

Based on these findings, our results suggest that the deployment of a capital-intensive SI technology, such as ZT, through private-sector SPs can lead to relatively socially inclusive outcomes as farmers’ awareness and trust in the technology increases and the service economy matures. Given the proven benefits of ZT for sustainable wheat intensification and poverty reduction in the Eastern IGP, the State Departments of Agriculture and State Agricultural Universities in Bihar and adjacent states should continue to strongly support its diffusion. To enhance farmers’ access to high-quality ZT services, institutional arrangements are needed to promote ZT service provision as a business opportunity and for the deployment of respective technical and, ideally, business development training to interested farmers. Targeting local dealers of ZT drills as a conduit for technical training appears to be a promising pathway; as buyers of ZT drills rarely receive any training from machine dealers thus far, this will require sensitizing dealers about the potential benefits for their own businesses in terms of a growing demand for ZT drills as farmers benefit from quality ZT services and share their experience with others.

From the case of ZT diffusion in Bihar, the following general lessons can be deduced for SI related research and extension efforts in developing countries:

*Research:* there tends to be a high pressure on R4D projects to quickly demonstrate impact. However, a premature assessment of the livelihood impacts of an intervention may yield misleading results; this is particularly true with respect to the social distribution of such impacts. Early adopters of agricultural innovations tend to systematically differ from later adopters (Rogers, 2003). The more knowledge-intensive a technology, and the more drastically it deviates from farmers’ traditional practices, the longer is the lag to be expected between early field testing by few ‘progressive’ farmers and widespread adoption; likewise, early testers may decide to discontinue the practice after some time. SI technologies, such as ZT, tend to be both knowledge-intensive and often constitute a major change in farmers’ management practices; hence, to realistically assess their livelihood impacts and social inclusiveness, an adequate time lag after the target population’s first exposure to these technologies needs to be allowed for. Further, market-based impact pathways such as mechanized service provision take time to mature with competition among providers increasing inclusion. The collection of panel data is instrumental for documenting and understanding these processes.

Detailed panel data such as the ones used for this study are costly to collect, and a more qualitative approach that seeks to collect information from change agents at regular intervals may provide a practical alternative method. For instance, if service providers of SI technologies can be identified by key informants or through snowball sampling techniques at the village level, a survey of (relatively few) service providers rather than a (relatively large) random sample of service users can provide important insights regarding technology uptake. Eliciting information about the average area covered per customer may yield proxy information about the development of the social inclusiveness of the practice as well. However, unlike the econometric analysis employed in this study, a qualitative approach is limited in its capacity to establish causality between technology adoption and its (potential) drivers, and to extrapolate findings to an underlying population.

*Extension:* to accelerate the uptake of SI technologies, development initiatives should make use of social networks for within-village information diffusion. As farmers usually interact with farmers of similar socioeconomic status, extension messages should be targeted to farmers representing different social strata. To scale capital-intensive mechanization technologies among smallholder farmers, extension efforts should emphasize business development for custom-hiring services to enable socially inclusive technology access. In addition, if such technologies are knowledge-intensive, technical and business development training needs to be systematized and led by institutions with aligned mandates and incentives. In the South Asia context, the private sector (e.g. machine dealers) appears poised to assume this responsibility where markets are either strong or promising. Quality custom-hiring services will help smallholders reap the full benefit from mechanized SI technologies, which will encourage their sustained and widespread adoption and ultimately benefit all actors along the supply chain.

## Declaration of Competing Interest

None.

## Figures and Tables

**Table A1:** Maximum Likelihood estimates of Heckman probit selection models explaining awareness of zero-tillage (ZT) and adoption of ZT wheat conditional on awareness based on panel data from *rabi* seasons 2012/13 and 2015/16 in Bihar (pooled model); coefficients are marginal effects.

	Awareness		Adoption cond. on awareness	
Variable	Coefficient^1^	z-value^2^	Coefficient^1^	z-value^2^
Rabi 2015/16	0.2706	11.05^****^	−0.0834	−1.63
Cultivable area	0.1220	3.37^***^	0.0891	3.31^***^
Cultivable area, sqd.	−0.0120	−2.17^**^	−0.0153	−3.62^****^
Maximum plot size	0.0079	0.27	0.0860	3.06^***^
Land owned^d^	−0.0118	−0.36	0.0186	0.54

Labor/land ratio	−0.0020	−3.51^****^	−0.0017	−1.55
Age	−0.0011	−1.31	−0.0004	−0.54
High education^d^	0.0438	1.28	0.0519	1.35
SC/ST category^d^	−0.0171	−0.50	−0.0562	−1.14
General caste category^d^	0.0092	0.34	0.1052	2.50^**^
Risk preference	0.0186	3.42^***^	0.0191	2.25^**^

Credit access	0.0014	0.55	−0.0047	−1.18

Farmer association^d^	0.0809	1.54	0.0877	1.34
Extension access	0.0143	1.89^*^	−0.0128	−1.56
Mobile phone^d^	−0.0066	−0.15	0.0214	0.28
Radio^d^	0.0807	3.29^***^	–	
TV^d^	0.0427	2.04^**^	–	
No ZT SP in 5 km^d^	0.0059	0.23	−1.3246	−10.51^****^

NM ZT use*smallest	0.0023	5.49^****^	0.0027	6.18^****^
NM ZT use*middle	0.0021	4.69^****^	0.0025	6.42^****^
NM ZT use*largest	0.0013	2.82^***^	0.0015	3.43^***^
NM meet frequency	0.0019	1.10	−0.0007	−0.39
NM age	−0.0012	−1.24	−0.0011	−0.70
NM education	0.0032	3.27^***^	0.0013	0.85
NM higher caste^d^	−0.0206	−0.50	0.0682	1.74^*^

Begusarai^d^	−0.2314	−6.40^****^	−0.1254	−3.22^***^
Buxar^d^	−0.0873	−2.30^**^	0.1962	3.97^****^
Lakhisarai^d^	−0.0660	−2.22^**^	0.1947	3.98^****^
Samastipur^d^	−0.1230	−3.95^****^	−0.3370	−4.18^****^
Vaishali^d^	−0.2656	−4.96^****^	−0.3936	−5.65^****^

N =	1846		1201	
Log pseudolikelihood = −1394.70				
Wald test of independent equations: chi-square (1) = 12.63^****^				

^*^(^**^)[^***^]{^****^} Significant at the 10%(5%)[1%]{0.1%} level of alpha error probability.

1 Coefficients are marginal effects (evaluated at means of all explanatory variables); for dummy variables, marginal effects are for a discrete change from 0 to 1.

2 Based on robust standard errors adjusted for 40 village-level clusters.

d Dummy variable.

**Table A2:** Maximum Likelihood estimates of a multinomial logit model explaining different categories of zero-tillage (ZT) wheat adoption in Bihar (definitions below the table); coefficients are relative probability ratios compared to ‘Non-adoption’ (N = 424) as base category.

	Early adoption (N = 182)		Recent adoption (N = 181)		Dis-adoption (N = 107)	
Variable	Coefficient^1^	z-value^2^	Coefficient^1^	z-value^2^	Coefficient^1^	z-value^2^
Cultivable area	2.5457	2.19^*^	0.915	−0.2	0.8315	−0.44
Cultivable area, sqd.	0.8801	−2.31^*^	0.9336	−1.11	1.0301	0.56
Maximum plot size	1.2802	0.77	2.0062	2.18^*^	1.0037	0.01
Land owned^d^	0.5617	−1.22	0.6667	−1.07	1.6978	0.99

Labor/land ratio	0.9564	−1.57	0.9845	−1.77	0.9533	−2.89^**^
Age	0.9907	−0.9	0.99	−0.92	0.9918	−0.92
High education^d^	2.4598	3.02^**^	1.3383	0.78	1.371	0.79
SC/ST category^d^	0.6262	−1.06	0.5037	−2.31^*^	0.4348	−1.92
General caste category^d^	2.1323	1.66	1.6957	1.26	1.0115	0.03
Risk preference	1.3018	2.54^*^	1.1366	1.21	0.9321	−0.72

Credit access	1.1869	1.15	1.2265	1.34	0.8962	−0.75

Farmer association^d^	3.289	1.6	2.186	1	1.8698	0.83
Extension access	0.9735	−0.38	0.9164	−0.91	1.0904	1.06
Mobile phone^d^	1.7136	0.46	0.6751	−0.68	0.4413	−1.18
Radio^d^	1.7887	1.04	2.7022	1.8	1.8846	1.52
TV^d^	1.2	0.62	1.3139	1.06	1.358	1.01
No ZT SP in 5 km^d^	0	−5.86^***^	0	−6.10^***^	0	−12.12^***^

NM ZT use*smallest	1.029	4.57^***^	1.0335	5.24^***^	1.0081	1.37
NM ZT use^*^middle	1.0377	6.96^***^	1.0393	7.30^***^	1.0165	3.77^***^
NM ZT use*largest	1.0272	4.77^***^	1.0341	5.79^***^	1.0134	2.54^*^
NM meet frequency	0.9644	−1.43	1.0218	0.97	0.9904	−0.29
NM age	1.0094	0.58	1.0036	0.23	0.988	−0.71
NM education	1.0176	0.8	0.9994	−0.03	1.0019	0.16
NM higher caste^d^	0.4879	−1.71	1.4798	0.83	0.9151	−0.17
Begusarai^d^	2.884	1.87	1.7692	1.15	1.1334	0.16
Buxar^d^	10.7556	3.66^***^	25.9547	3.97^***^	1.0242	0.03
Lakhisarai^d^	0.2934	−3.08^**^	0.2907	−2.73^**^	1.0028	0.01
Samastipur^d^	0.0098	−4.23^***^	0.0069	−8.07^***^	0.3464	−1.59
Vaishali^d^	0.0156	−3.43^***^	0.0093	−3.97^***^	0.4104	−1

N =	894					
Log pseudolikelihood =	−760.32					
Pseudo R^2^ =	0.323					

^*^(^**^)[^***^]{^****^} Significant at the 10%(5%)[1%]{0.1%} level of alpha error probability.

^1^Coefficients are exponentiated value of parameter estimates from mlogit model. It represents the relative risk ratio for a one-unit change in the corresponding variable (for dummy variables it is a change from 0 to 1), where risk is measured as the risk of the outcome relative to the base outcome.

^2^Based on robust standard errors adjusted for 40 village-level clusters.

^d^Dummy variable.

Definitions:

Early adoption = continuous use of ZT since 2012 or earlier (data available until 2010).

Recent adoption = continuous use of ZT since 2013 or later (data available until 2015).

Non-adoption = never used ZT throughout the period 2010–2015.

Dis-adoption = used ZT earlier, but did not use it for at least three consecutive years and/or stated that they stopped using ZT.

**Table A3:** Basic respondent household (HH) characteristics, differentiated by zero-tillage (ZT) adoption status (values are means unless indicated otherwise).

Respondent characteristics	(1) Cultivable area (ha)	(2) Size of largest irrigable plot (ha)	(3) Labor-to-land ratio	(4) Years of education of HH head	(5) % HH heads belonging to Scheduled castes	(6) % HH heads belonging to General caste	(7) No. of ZT service providers in 2 km radius	(8) No. of ZT service providers in 5 km radius	(9) % HHs with no ZT service provider in 5 km radius	(10) % ZT adoption rate among NMs^1^
Early adopters (N = 182)	2.06^a^	1.03^a^	4.10^a^	9.43^a^	8.2	56.6	4.24^a^	35.35^a^	0.0	70.1^a^
Recent adopters (N = 181)	1.26^bc^	0.73^b^	7.69^b^	7.63^bc^	12.2	36.5	4.35^a^	34.52^a^	0.0	74.6^a^
Dis-adopters (N = 107)	1.42^b^	0.60^b^	6.14^b^	8.58^ac^	8.4	42.1	2.51^b^	27.60^b^	0.0	45.3^b^
Non-adopters (N = 424)	1.07^c^	0.44^c^	11.24^b^	6.73^b^	15.6	35.6	2.50^b^	24.00^b^	4.7	26.3^c^
Stat. sig.	^*^^2^	^*^^2^	^***^^2^	^**^^2^	^*^^3^	^***^^3^	^***^^2^	^*^^2^	^***^^3^	^***^^2^
Whole sample (N = 894)	1.35	0.62	8.46	7.46	12.5	40.8	3.23	28.88	2.2	47.3

^*^(^**^)[^***^] Significant at the 5% (1%)[0.1%] level of alpha error probability.

1 Based on three primary network members (NMs); only ZT adoption prior to the respondent is considered.

2 Based on (multiple) Mann-Whitney tests, accounting for family-wise error; diverging superscript letters indicate statistical significance *at least* at the indicated level.

3 Based on chi-square test.

## Footnotes

1 Based on a random sample of ZT service providers in Bihar, tractors averaged 38.3 HP in 2012 (Keil et al., 2016).

2 Based on village survey data collected in 2008.

3 For simplification, in the following we refer to the year when wheat was sown rather than harvested, i.e. 2012 refers to *rabi* season 2012/13 etc.

4 Household heads were male in 98% of cases. Due to the non-sensitive nature of the research topic, the male enumerators did not face any problem in interviewing the few female household heads. The enumerators worked for a New Delhi-based consulting firm specialized in research and communications in the social and agricultural sectors.

5 Note that a panel regression based on differences in values of dependent and independent variables over time has severe limitations in binary dependent variable models; in our case, both-period adopters would be treated the same as both-period non-adopters.

6 The maximum likelihood procedure does not directly estimate ρ but $atanh\rho =\frac{1}{2}\ln\left(\frac{1+\rho }{1-\rho }\right)$.

7 This procedure was used to replace a Chow test which is applicable to linear models only.

8 Based on the conventionally used cut-off probability of 50%.

9 Condition for maximum fulfilled for Cultivable area = 0.1478406/0.011797 = 12.53 ha (rounded coefficients are shown in Table 5).

10 An alternative specification omitting the squared term leads to a positive but insignificant coefficient of *Cultivable area* in the awareness stage and to a negative but insignificant coefficient in the adoption stage. For reasons of direct comparability with Model 1 we retained the squared term.

11 Irrigation is a prerequisite to growing wheat during *rabi* season in Bihar.

12 The estimated marginal effect exceeds the value of 1 in 2012 in absolute terms, which is likely due to the approximation method used in the estimation (Boggess and MacDonald, StataCorp; https://www.stata.com/support/faqs/statistics/marginal-effect-greater-than-1; accessed 01/11/2018). For 2015, the magnitude of the coefficient indicates a more plausible reduction of the probability of using ZT by 60 percentage points.

13 Including the mass media related dummies *Radio* and *TV* that were omitted from the adoption stage in the heckprobit model.

14 An alternative way of presenting the regression results is shown in Table A2 in the Annex, which displays relative probability ratios for a one-unit change in each explanatory variable compared to a base outcome (Non-adoption). We use the effect of *Max. plot size* on recent adoption to illustrate the difference in interpretation: Table 6 indicates that, taking all explanatory variables at their means, an increase in plot size by one hectare increases the *absolute* probability of being a recent adopter by 6.65 *percentage points*. Table A2 indicates that an increase in plot size by one hectare increases the probability of being a recent adopter *rather than a non-adopter* by 100.62 *percent*. While both tables lead to the same conclusions, we prefer to display absolute marginal effects for their more straight-forward interpretation.

## References

[b0005] Aggarwal P.K., Joshi P.K., Ingram J.S.I., Gupta R.K. (2004). Adapting food systems of the Indo-Gangetic plains to global environmental change: Key information needs to improve policy formulation. Environmental Science & Policy.

[b0010] Alexandratos N., Bruinsma J. (2012). World agriculture towards 2030/2050: The 2012 revision.

[b0015] Aryal J.P., Sapkota T.B., Jat M.L., Bishnoi D.K. (2015). On-farm economic and environmental impact of zero-tillage wheat: A case of North-West India. Experimental Agriculture.

[b0020] Chambers R., Conway G. (1992). Sustainable rural livelihoods: Practical concepts for the 21st century.

[b0025] Chauhan B.S., Mahajan G., Sardana V., Timsina J., Jat M.L. (2012). Productivity and sustainability of the rice-wheat cropping system in the Indo-Gangetic Plains of the Indian subcontinent: Problems, opportunities, and strategies. Advances in Agronomy.

[b0030] Dhiman S.D., Kumar S., Om H. (2003). Shallow tillage and drill technology for wheat. Indian Farming.

[b0035] Diagne A., Demont M. (2007). Taking a new look at empirical models of adoption: Average treatment effect estimation of adoption rates and their determinants. Agricultural Economics.

[b0040] DoA (2008). Bihar's Agricultural Development: opportunities and challenges – A report of the special task force on Bihar Department of Agriculture.

[b0045] Doss C.R. (2006). Analyzing technology adoption using microstudies: Limitations, challenges, and opportunities for improvement. Agricultural Economics.

[b0050] Erenstein O., Laxmi V. (2008). Zero-tillage impacts in India's rice-wheat systems: A review. Soil and Tillage Research.

[b0055] Feder G., Just R., Silberman D. (1985). Adoption of agricultural innovations in developing countries: A survey. Economic Development and Cultural Change.

[b0060] Feder G., Savastano S. (2006). The role of opinion leaders in the diffusion of new knowledge: The case of integrated pest management. World Development.

[b0065] Foster A.D., Rosenzweig M.R. (1995). Learning by doing and learning from others: Human capital and technical change in agriculture. Journal of Political Economy.

[b0070] Garnett T., Appleby M.C., Balmford A., Bateman I.J., Benton T.G., Bloomer P., Godfray H.C.J. (2013). Sustainable intensification in agriculture: Premises and policies. Science.

[b0075] Gathala M.K., Kumar V., Sharma P.C., Saharawat Y.S., Jat H.S., Singh M., Ladha J.K. (2013). Optimizing intensive cereal-based systems addressing current and future drivers of agricultural change in the northwestern Indo-Gangetic Plains of India. Agriculture, Ecosystems and Environment.

[b0080] Godfray H.C.J., Beddington J.R., Crute I.R., Haddad L., Lawrence D., Muir J.F., Toulmin C. (2010). Food security: The challenge of feeding 9 billion people. Science.

[b0085] Granovetter M.S. (2005). The impact of social structure on economic outcomes. Journal of Economic Perspectives.

[b0090] Hausman J., McFadden D. (1984). Specification tests for the multinomial logit model. Econometrica.

[b0095] Heckman J.J. (1979). Sample selection bias as a specification error. Econometrica.

[b0100] Humphreys E., Kukal S.S., Christen E.W., Hira G.S., Singh B., Yadav S., Sharma R.K. (2010). Halting the groundwater decline in North-West India – which crop technologies will be winners?. Advances in Agronomy.

[b0105] Hunter M.C., Smith R.G., Schipanski M.E., Atwood L.W., Mortensen D.A. (2017). Agriculture in 2050: Recalibrating targets for Sustainable Intensification. BioScience.

[b0110] Kabunga N.S., Dubois T., Qaim M. (2012). Heterogeneous information exposure and technology adoption: The case of tissue culture bananas in Kenya. Agricultural Economics.

[b0115] Keil A., D'souza A., McDonald A.J. (2015). Zero-tillage as a pathway for sustainable wheat intensification in the Eastern Indo-Gangetic Plains: Does it work in farmers’ fields?. Food Security.

[b0120] Keil A., D'souza A., McDonald A.J. (2016). Growing the service economy for sustainable wheat intensification in the Eastern Indo-Gangetic Plains: Lessons from custom hiring services for zero-tillage. Food Security.

[b0125] Keil A., D'souza A., McDonald A.J. (2017). Zero-tillage is a proven technology for sustainable wheat intensification in the Eastern Indo-Gangetic Plains: What determines farmer awareness and adoption?. Food Security.

[b0130] Keil A., Mitra A., D'souza A., McDonald A.J. (2018). Assessing the adoption dynamics of zero-tillage (ZT) wheat and the growth dynamics of related custom hire services in Bihar. Survey 1: ZT adoption and its welfare impacts at the farm household level. http://hdl.handle.net/11529/10548164.

[b0135] Keil A., Mitra A., D'souza A., McDonald A.J. (2018). Assessing the adoption dynamics of zero-tillage (ZT) wheat and the growth dynamics of related custom hire services in Bihar. Survey 2: ZT service provision as a business opportunity. http://hdl.handle.net/11529/10548165.

[b0140] Krishna V.V., Keil A., Aravindakshan S., Meena M., Langridge P. (2017). Conservation tillage for sustainable wheat intensification in South Asia. Achieving sustainable cultivation of wheat, Volume 2: Cultivation techniques.

[b0150] Loos J., Abson D.J., Chappell M.J., Hanspach J., Mikulcak F., Tichit M., Fischer J. (2014). Putting meaning back into “sustainable intensification”. Frontiers in Ecology and the Environment.

[b0155] Manski C.F. (1993). Identification of endogenous social effects: The reflection problem. Review of Economic Studies.

[b0160] Manski C.F. (2000). Economic analysis of social interactions. Journal of Economic Perspectives.

[b0165] Matuschke I., Qaim M. (2009). The impact of social networks on hybrid seed adoption in India. Agricultural Economics.

[b0170] Mehla R.S., Verma J.K., Gupta R.K., Hobbs P.R. (2000). Stagnation in the productivity of wheat in the Indo-Gangetic Plains: Zero-Till-Seed-Cum-Fertilizer Drill as an integrated solution.

[b0175] MoA (2017). Agricultural Statistics at a Glance 2016.

[b0180] Rasmussen L.V., Coolsaet B., Martin A., Mertz O., Pascual U., Corbera E., Ryan C.M. (2018). Social-ecological outcomes of agricultural intensification. Nature Sustainability.

[b0185] Rogers E.M. (2003). The diffusion of innovations.

[b0190] Scoones I. (1998). Sustainable rural livelihoods: A framework for analysis.

[b0195] Singh R., Erenstein O., Saharawat Y.S., Chaudhary N., Jat M.L. (2012). Adoption analysis of resource-conserving technologies in rice (Oriza sativa) – wheat (Triticum aestivum) cropping system of South Asia. Indian Journal of Agricultural Sciences.

[b0200] Singh A., Phogat V.K., Dahiya R., Batra S.D. (2014). Impact of long-term zero till wheat on soil physical properties and wheat productivity under rice-wheat cropping system. Soil & Tillage Research.

[b0205] Songsermsawas T., Baylis K., Chhatre A., Michelson H. (2016). Can peers improve agricultural revenue?. World Development.

[b0215] Tilman D., Balzer C., Hill J., Befort B.L. (2011). Global food demand and the sustainable intensification of agriculture. Proceedings of the National Academy of Sciences of the U.S.A. (PNAS).

[b0220] Tobin J. (1958). Estimation of relationships for limited dependent variables. Econometrica.

[b0225] United Nations General Assembly (2005). 2005 World Summit Outcome, Resolution A/60/1. http://data.unaids.org/topics/universalaccess/worldsummitoutcome_resolution_24oct2005_en.pdf (accessed on 07/27/2018).

[b0230] van de Ven W.P.M.M., van Praag B.M.S. (1981). The demand for deductibles in private health insurance. Journal of Econometrics.

[b0235] Wooldridge J.M. (2006). Introductory econometrics. A modern approach.

